# Proactive inhibition is not modified by deep brain stimulation for Parkinson's disease: An electrical neuroimaging study

**DOI:** 10.1002/hbm.25530

**Published:** 2021-06-10

**Authors:** Michael De Pretto, Michael Mouthon, Ines Debove, Claudio Pollo, Michael Schüpbach, Lucas Spierer, Ettore A. Accolla

**Affiliations:** ^1^ Neurology Unit, Medicine Section, Faculty of Sciences and Medicine University of Fribourg Fribourg Switzerland; ^2^ Movement Disorders Center, Department of Neurology, Inselspital Bern University Hospital, University of Bern Bern Switzerland; ^3^ Department of Neurosurgery Inselspital University Hospital Bern Bern Switzerland; ^4^ Neurology Unit, Department of Medicine HFR – Cantonal Hospital Fribourg Fribourg Switzerland

**Keywords:** EEG, indirect pathway, inhibitory control, internal globus pallidus, subthalamic nucleus

## Abstract

In predictable contexts, motor inhibitory control can be deployed before the actual need for response suppression. The brain functional underpinnings of proactive inhibition, and notably the role of basal ganglia, are not entirely identified. We investigated the effects of deep brain stimulation of the subthalamic nucleus or internal globus pallidus on proactive inhibition in patients with Parkinson's disease. They completed a cued go/no‐go proactive inhibition task ON and (unilateral) OFF stimulation while EEG was recorded. We found no behavioural effect of either subthalamic nucleus or internal globus pallidus deep brain stimulation on proactive inhibition, despite a general improvement of motor performance with subthalamic nucleus stimulation. In the non‐operated and subthalamic nucleus group, we identified periods of topographic EEG modulation by the level of proactive inhibition. In the subthalamic nucleus group, source estimation analysis suggested the initial involvement of bilateral frontal and occipital areas, followed by a right lateralized fronto‐basal network, and finally of right premotor and left parietal regions. Our results confirm the overall preservation of proactive inhibition capacities in both subthalamic nucleus and internal globus pallidus deep brain stimulation, and suggest a partly segregated network for proactive inhibition, with a preferential recruitment of the indirect pathway.

## Graphical abstract

Motor inhibition allows for a flexible control of goal‐directed behaviour. We report an overall preservation of proactive inhibition capacities with deep brain stimulation in Parkinson's patients, and suggest a partly segregated brain network for proactive inhibition.

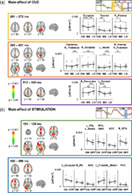

## 1 INTRODUCTION

Motor inhibitory control is a fundamental capacity involved in controlling behaviour, when external environmental stimuli prompt the suppression of ongoing actions. Importantly, the efficacy of motor inhibition is modulated by context: if an interruption of the action is expected, it will be more readily deployed because the inhibition would have been prepared. Referred to as ‘proactive’ inhibition, the readiness to suppress motor action might for instance allow for an easier interruption of walking when the switch between a green to a red traffic light is preceded by a flashing phase. Yet, while proactive inhibition better reflects daily life situation that reactive control, its neural underpinning remains underexplored (Stuphorn & Emeric, 2012).

Current evidence indicate that proactive inhibitory control involves a brain network partly overlapping with reactive control, namely the pre‐supplementary motor area, pre‐SMA, and the right inferior frontal gyrus (rIFG), as well as the subthalamic nucleus (STN; Aron, 2011; Aron, Behrens, Smith, Frank, & Poldrack, 2007a; Aron & Poldrack, 2006; van Belle, Vink, Durston, & Zandbelt, 2014; Zandbelt, Bloemendaal, Neggers, Kahn, & Vink, 2013). The pre‐SMA and IFG first react to stopping stimuli, followed by an early recruitment of STN through the ‘hyperdirect’ pathway (Aron, Robbins, & Poldrack, 2014).

While some authors advance that proactive inhibition depend merely on the modulation of the hyperdirect pathway (Stuphorn & Emeric, 2012), other accounts propose that the ‘indirect pathway’ has a specific role in this process. This latter view implies a more important role for the dorsolateral prefrontal cortex (DLPFC) and the caudate, external and internal globus pallidum in proactive response suppression (Jahanshahi, Obeso, Rothwell, & Obeso, 2015). Yet, the involvement of each these structure remains largely speculative.

Deep brain stimulation of the STN improves motor symptoms of Parkinson's disease interfering with dysfunctional oscillations within basal ganglia circuitry, eventually favouring a prokinetic state (Chiken & Nambu, 2016). Despite the proposed centrality of STN in inhibitory control, the few studies having investigated the effects of STN‐DBS on proactive inhibition reported no effect (Mancini et al., 2019; Mirabella et al., 2012), or even suggested a possible normalisation of proactive capabilities (Obeso, Wilkinson, Rodríguez‐Oroz, Obeso, & Jahanshahi, 2013). Experimental design is probably key for capturing subtle modifications. For instance, Mirabella et al. (2013) could demonstrate that STN‐DBS restores to normal the relationship between reaction time and movement time in the context of uncertainty, possibly modulating proactive inhibition mechanisms still active during movement execution. Likewise, comparisons of the effects of dopaminergic drugs and STN‐DBS suggest that STN‐DBS may specifically normalise the capacity of releasing proactive inhibition, contrary to dopaminergic drug treatment (Favre, Ballanger, Thobois, Broussolle, & Boulinguez, 2013).

The internal globus pallidus (GPi) is a more rarely chosen target for DBS in Parkinson's disease. Despite allowing for a lesser decrease of dopaminergic medication than STN‐DBS, GPi‐DBS has a comparable efficacy on motor symptoms, and in the reduction of fluctuations and dyskinesia (Williams, Foote, & Okun, 2014). As a common structure within multiple basal ganglia pathways (direct, indirect, hyperdirect), it is involved in inhibitory control, but has been less studied than STN. Current evidence suggest a positive effect of GPi‐DBS in triggering the action, with no impairment in its suppression (Kohl et al., 2015), or even an improvement of the dysfunctional proactive inhibition after turning GPi stimulator ON (Pan et al., 2018).

In the present study, we aimed at resolving the inconsistencies of previous literature and examined thoroughly the involvement of STN and of the internal globus pallidus (GPi) in proactive inhibition. To this aim, we capitalised on Parkinson's disease patients previously operated for deep brain stimulation (DBS) of either of the two nuclei.

We developed a task requiring the deployment of varying levels of proactive inhibitory control, and studied the behavioural and electrophysiological effects of the stimulation of STN and GPi in Parkinson's Disease patients previously operated for DBS. Topographic and source estimation analyses of event‐related potentials recorded between the presentation of a cue and the Go/NoGo probe were conducted to examine the functional effect of the stimulation. Turning the device on and off, we contrasted the electrophysiological activity associated with the engagement of proactive inhibition in the two stimulation conditions.

Given the above, we expected (a) that both STN‐ and GPi‐DBS reduce an excessive proactive inhibition, related to PD symptoms, (b) that both STN‐ and GPi‐DBS decrease the capacity of modulating proactive inhibition, interfering with the activity of the hyperdirect pathway and the indirect pathway, respectively; and (c) EEG evidence of the involvement of DLPFC, IFG, and pre‐SMA during the task.

## 2 METHODS

### 2.1 Participants

We recruited patients with Parkinson's disease from the Neurological Department, Movement Disorders Unit, Bern University Hospital, and from the Neurology Department, Fribourg Cantonal Hospital, Switzerland.

The cohort included 12 non‐operated patients (NO), 14 STN‐DBS patients (STN), and seven GPi‐DBS patients (GPi). All patients signed an informed consent according to the Declaration of Helsinki and to the protocol approved from the local ethics committee (Protocol PB_2016‐01384). Five NO patients were excluded from the analyses: two were suspected of drug‐induced parkinsonism, one was not able to complete the task, and two had excessively noisy EEG signal resulting in too few ERP trials for averaging. As we focused on the ERPs time‐locked to the cue and thus not contaminated by the activity related to the motor response, we did not exclude left‐handed patients from the EEG analysis (two NO and two STN). In the STN group, one left‐handed participant was tested as a right‐handed. The second left‐handed participant was tested on the left side and thus, the stimulator was switched off on the opposite side as the other participants. As the analyses with and without this patient showed the same results, we kept this patient to maximise our statistical power. Previous studies have shown that reactive and proactive inhibition behavioural performance was not influenced by the hand employed to perform the task (Caprio, Modugno, Mancini, Olivola, & Mirabella, 2020; Mirabella, Fragola, Giannini, Modugno, & Lakens, 2017).

Demographic data are summarised in Table 1. Hoehn and Yahr staging was not assessed formally but we estimate it was for all patients in all groups between II and III. Group comparisons were performed using non‐parametric Kruskal–Wallis One‐Way ANOVAs.

**TABLE 1:** Demographic data

	NO	STN	GPi	*p*‐value	NO vs STN	NO vs GPi	STN vs GPi
*n*	12	14	7				
Female [%]	25%	36%	57%	.383			
Age [mean (sd)]	62.1 (11.2)	64.3 (9.9)	66.4 (4.4)	.538			
Disease duration [mean (sd)]	4.1 (2.8)	14.6 (6.1)	17.5 (7.5)	.000	0.000	0.005	0.786
Time since operation [mean (sd)]	n/a	1.9 (3)	2 (2.9)	.961			
UPDRS ON [mean (sd)]	22.3 (3.9)	17 (6.3)	25 (6.5)	.016	0.038	0.738	0.075
UPDRS OFF [mean (sd)]	n/a	25.1 (5.4)	25.4 (6.1)	1.000			
QUIP [mean (sd)]	21.6 (20.9)	8.8 (12.4)	15 (16.5)	.211			
BDI [mean (sd)]	8.9 (6.1)	4 (2.9)	6.3 (4.1)	.029	0.023	0.698	0.341
MoCA [mean (sd)]	25.7 (2.6)	27.1 (1.5)	26.7 (2.7)	.441			
LEDD [mean (sd)]	575.0 (397.1)	401.7 (440.2)	1,057.2 (392.4)	.007	0.355	0.033	0.016

Abbreviations: BDI, Beck Depression Inventory; LEDD, Levodopa Equivalent Daily Dose; MoCA, Montreal Cognitive Assessment; QUIP, Questionnaire for Impulsive‐Compulsive Disorders in Parkinson's Disease; UPDRS, Unified Parkinson's Disease Rating Scale.

We collected clinical and demographic information from the patients before the experiment and during the 30 min pause between sessions. Patients filled in questionnaires assessing mood (Beck Depression Inventory, BDI); and the presence and severity of impulse control disorders (Questionnaire for Impulsive‐Compulsive Disorders in Parkinson's Disease, QUIP, (Weintraub et al., 2012). They underwent a Montreal Cognitive Assessment (MoCA) conducted by an experienced neurologist or neuropsychologist during the experimental session, if not available from medical records and not older than 3 months. All patients were examined by a trained neurologist (EAA, ID) for assessing the motor part of the Movement Disorders Society Unified Parkinson's Disease Rating Scale (0–132 points, MDS‐UPDRS III, [Goetz et al., 2008]). All patients were tested under their usual antiparkinsonian medication.

### 2.2 Stimuli and task

**FIGURE 1:**
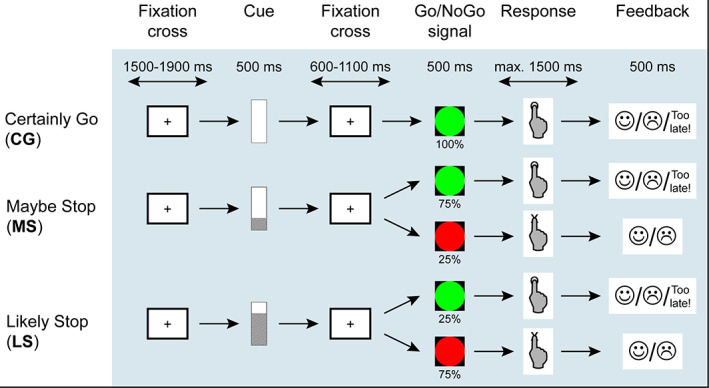
Experimental task. The patients had to respond as fast as possible to the Go stimuli (green circle) while withholding their responses to the NoGo stimuli (red circles). A Cue preceding the Go/NoGo signals indicated the probability of a NoGo signal to occur

The cued Go/NoGo task involved a green circle as Go signal and a red circle as NoGo signal preceded by a cue informing on the probability of an upcoming NoGo signal (Figure 1). The cue was represented by a vertical bar, in three alternative configurations. If the bar was completely white, then there was a 0% chance that the following trial would be a NoGo. If the bar was only partially shaded, then there was a 25% chance that the following trial would be a NoGo. This probability reached 75% if the symbol was almost all shaded. The participants were not informed about the detailed probabilities, but only given general indications (‘certainly go’, ‘maybe stop’, ‘likely stop’).

Each trial started with a white fixation cross on light grey background (1500–1900 ms). During fixation, the participants were asked to keep their hand on a response box (E‐Prime Chronos box), with the index finger ready to press the key. The cue was then presented for 1,000 ms. The delay between the cue and the probe was variable (600–1,100 ms) to reduce the predictability of the task. Participants were instructed to press the key as fast as possible as soon as they saw a Go signal, and to withhold their response in case of a NoGo signal. They were shown a brief example in order to familiarise them with the task and stimuli. Then, they completed a short training session of 12 trials during which we record each participant's average reaction time. This mean value was used as a threshold during execution of the task. If their response to Go trials exceeded the threshold, they saw a ‘too late!’ feedback message. This added time pressure to encourage faster reaction times (De Pretto, Rochat, & Spierer, 2017; Vocat, Pourtois, & Vuilleumier, 2008). The ‘too late’ trials were included in the average RTs so that the RT distribution's right tail was preserved (Mancini, Falciati, Maioli, & Mirabella, 2020). Alternatively, they received a feedback on whether they performed the correct action, with a pictorial face with happy or sad expressions. The participants completed two sessions of three consecutive blocks. Each block consisted of 36 randomly presented trials (12 ‘certainly go’, 12 ‘maybe stop’, 12 ‘likely stop’), for a total of 24 Go trials (12 following a ‘certainly go’ cue, 9 ‘maybe stop’, 3 ‘likely stop’), and 12 NoGo trials (0 following a ‘certainly go’ cue, 3 ‘maybe stop’, 9 ‘likely stop’).

DBS‐operated patients (STN and GPi) completed one session with their stimulator on (bilateral) and one session after turning off the stimulation contralateral to the task performing hand. The two sessions were separated by a 30‐min break, allowing after‐effects from the previous stimulation to wane. The order of the stimulator sessions (ON or OFF) was randomised to control for learning effects. For non‐operated patients, the dominant and non‐dominant hands were both tested, always starting with the dominant hand side (to match stimulated patients, always tested on the dominant side). UPDRS‐III score was calculated before each session in DBS patients.

### 2.3 EEG recording and pre‐processing

Electroencephalogram (EEG) was recorded at a sampling rate of 1,024 Hz over 64 channels following the extended 10–20 system, with a Biosemi ActiveTwo system (Biosemi, Amsterdam, Netherlands). Offline pre‐processing of the raw EEG signal was conducted using in‐house Matlab scripts and EEGlab (Delorme & Makeig, 2004), in order to obtain ERPs time‐locked to the cue, over all electrodes.

Raw EEG data were first filtered using a 0.5–40 Hz band‐pass, which removed DBS‐related high frequency noise (Lio, Thobois, Ballanger, Lau, & Boulinguez, 2018; Sun et al., 2014). Removal of occasional, large amplitude non‐brain noise such as eye and muscle artefacts was completed with the Artefact Subspace Reconstruction EEGlab plugin (ASR; Chang, Hsu, Pion‐Tonachini, & Jung, 2018; Mullen et al., 2015). The EEG signal was then segmented into epochs from 100 ms pre‐cue onset to 600 ms post‐cue onset, with a baseline correction applied over the whole epoch window. Due to remaining eye blinks in the GPi group, individual data for this group went through an ICA procedure using the AMICA algorithm with default settings (Hsu et al., 2018; Palmer, Makeig, Kreutz‐Delgado, & Rao, 2008). This step was not necessary in the NO and STN groups. Channels showing a bad signal were interpolated (mean: 2.3 channels) using multiquadric interpolation relying on radial basis functions (Buhmann & Jäger, 2019; Jäger, Klein, Buhmann, & Skrandies, 2016). Epochs with at least one time‐frame ±80 μV were automatically rejected and the remaining epochs were averaged across trial for each cue separately. Finally, ERPs were re‐referenced to the common average reference.

The number of accepted epochs for each condition were controlled in order to ensure that any observed between‐condition differences were not due to differences in signal‐to‐noise ratio (STN: 35.7 ± 0.5, *p* = .630; GPi: 33.5 ± 4.8, *p* = .613; NO: 35.7 ± 1.0, *p* = .523).

### 2.4 Data analysis

Because of the heterogeneity of the groups (NO had no stimulation, GPi was limited in size), we analysed the three groups separately. For all statistical tests, our alpha threshold was set at 0.05, and effect sizes are reported for behavioural data.

### 2.5 Behavioural analysis

Performance at the Go/NoGo task was assessed by extracting the response times (RT) to Go stimuli and the false alarm rate (FA) to NoGo stimuli, that is, the percentage of inaccurately responded NoGo trials. We removed trials with RTs ≤ 100 ms as they reflect implausible cognitive processing of the Go signal (Gabay & Behrmann, 2014). We then identified univariate outliers using the median absolute deviation (MAD; Leys, Ley, Klein, Bernard, & Licata, 2013), with the suggested default parameters (i.e., MAD range around the median of 1.48 and level of decision of 2.5). Two participants were flagged as potential outliers for RT in one condition (LS‐OFF for an STN patient and MS‐ON a GPi patient). As they showed no other extreme values (either for RT or FA), we considered the flagged values as belonging to the distribution of interest and kept them in the dataset (Leys, Delacre, Mora, Lakens, & Ley, 2019).

RT and FA were averaged for each Cue type and Stim session (only the dominant hand session for the NO group). Normality of the data was assessed using the Shapiro–Wilk test a criterion of skewness and kurtosis within a ± 2 range (Kim, 2013). Whenever Mauchly's test indicated sphericity violation, we reported corrected *p*‐values using the Greenhouse–Geisser estimates.

RT in the STN and GPi groups was analysed using repeated measures ANOVAs (2 Stimulation [ON; OFF] × 3 Cue [CG; MS; LS]), and in the NO group using a one‐way repeated measures ANOVA (3 Cue [CG; MS; LS]). Because RT in the GPi group the assumption of normality was not consistently met, we verified the results using a Friedman non‐parametric repeated measures ANOVA. Given the low number of FA occurrences, we did not conduct statistical analyse over FA rates.

### 2.6 EEG analysis

All analyses were conducted using the Cartool software (Brunet, Murray, & Michel, 2011), the STEN toolbox developed by Jean‐François Knebel and Michael Notter (http://doi.org/10.5281/zenodo.1164038), and the RAGU toolbox developed by T. Koenig (Koenig, Kottlow, Stein, & Melie‐García, 2011). Because the signal at one electrode is the sum of the electrical activity from all over the brain, we used global measures of the electrical signal at each time point of the ERPs of all electrodes allowing neurophysiological interpretation of scalp‐recorded ERPs (Michel & Murray, 2012; Murray, Brunet, & Michel, 2008; Tzovara, Murray, Michel, & Lucia, 2012): (a) the global field power (GFP) is a measure of the global strength of the electric field, indexing modulation of response strength of the intracranial generators; (b) the global map dissimilarity (GMD) assesses the dynamic changes of scalp‐recorded electric field configuration (ERP topography) indexing modulations of the configuration of intracranial generators. In the cases of significant GMD and/or GFP effects, distributed electrical source estimation were computed and statistically compared to identify the brain generator underlying the effect measured at the scalp.

GFP and GMD were analysed in the RAGU software by computing time‐frame wise randomisation statistics. These analyses followed the same design as for RT: Stimulation [ON; OFF] × Cue [CG; MS; LS] repeated measures ANOVAs for the STN and GPi groups, and a 3 Cue [CG; MS; LS] one‐way repeated measures ANOVA for the NO group. The randomisation additionally estimates a minimal duration threshold for contiguous significant effects to account for multiple tests and temporal autocorrelation (Koenig et al., 2011). Only periods of statistical significance longer than the duration threshold were interpreted.

For each significant period identified, we averaged the individual ERP signals across timeframes and computed brain source estimation. For this, we applied a local autoregressive average (LAURA) distributed linear inverse solution (Grave de Peralta Menendez, Gonzalez Andino, Lantz, Michel, & Landis, 2001; Grave de Peralta Menendez, Murray, Michel, Martuzzi, & Gonzalez Andino, 2004) to the MNI average brain. Skull thickness and relative conductivity were estimated for a mean age of 65, as implemented in the Cartool software, yielding spatial gradient of current densities across neighbouring solution points. The solution space included 5,006 nodes equally distributed on a 6 × 6 × 6 mm grid within the grey matter of the Montreal Neurological Institute (MNI) average brain.

In order to identify brain areas associated with the effects observed at the ERP level, we applied the same design as for ERP analyses over each node using STEN. We corrected for multiple testing and spatial autocorrelation by applying a spatial‐extent threshold of at least 19 contiguous nodes with *p* < .05. This spatial criterion was calculated with the AlphaSim program (available from the Analysis of Functional NeuroImages website: http://afni.nimh.nih.gov). This program applies a cluster randomisation approach by computing 10,000 Monte Carlo permutations performed on our lead field matrix, assuming a spatial smoothing of 6 mm FWHM and a cluster connection radius of 8.5 mm. For clusters of at least 19 nodes, the output indicated a node‐level false positive probability of *p* < .001 for a cluster‐level likelihood of *p* < .05.

## 3 RESULTS

### 3.1 Behavioural results

**FIGURE 2:**
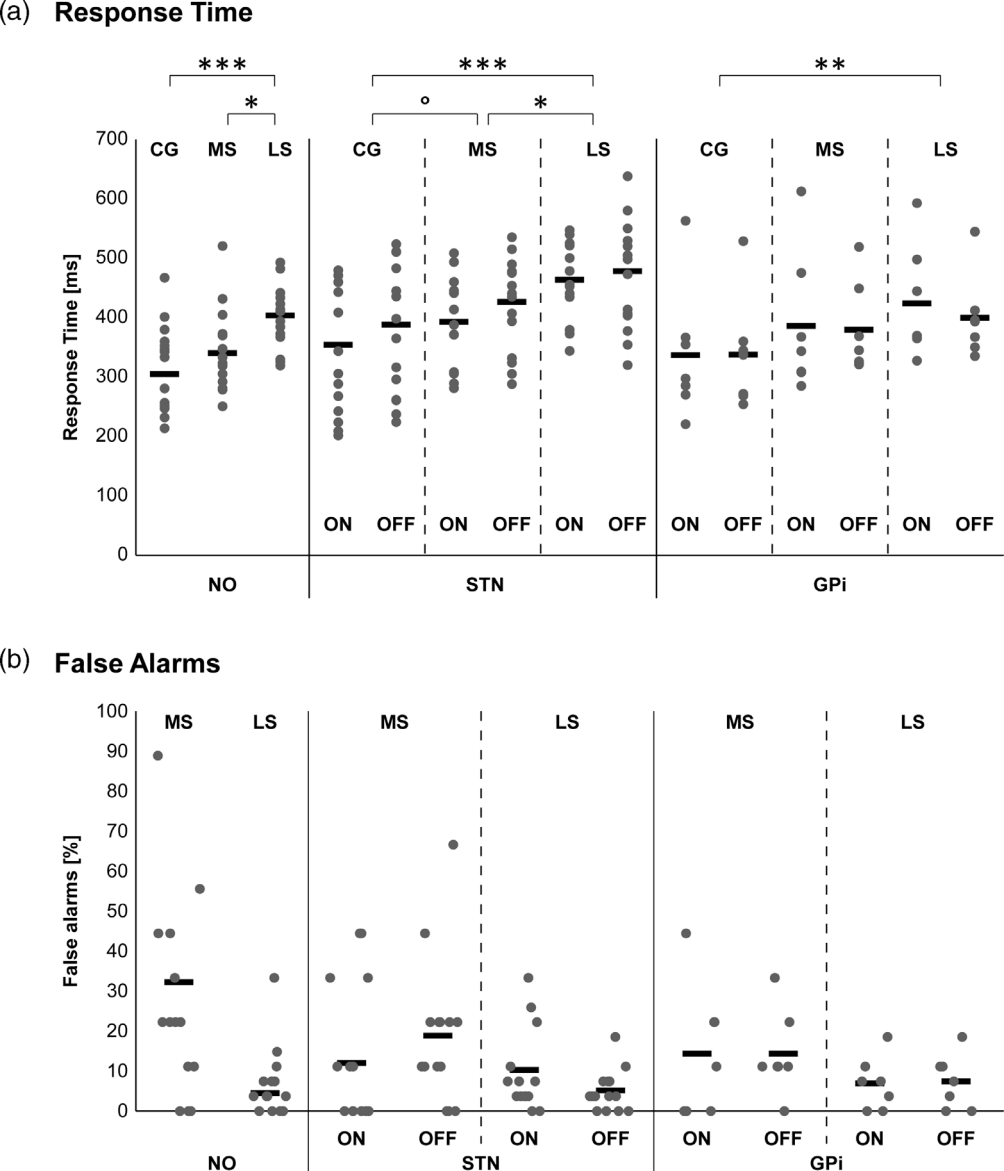
Behavioural results. Response times (a) and false alarm rates (b) for each group and condition. Thick horizontal lines represent the mean, and the grey dots represent the individual data. Post‐hoc *t* tests detailing the main effects of Cue are shown in A (Holm‐Bonferroni corrected). ***p < .001; **p < .01; *p < .05; °*p* = .051

The behavioural results are reported in Figure 2. Detailed behavioural results can be found in Table S1.

### 3.2 Response time

All groups showed a main effect of Cue (Table 2), driven by a gradual increase in RT as the probability of having a NoGo signal increased (Figure 2a). As the assumption of normality was not consistently met in the GPi group, we verified the result with a Friedman non‐parametric repeated measures ANOVA on the Cue factor. It also reached significance, *χ* ^2^(2) = 6.00, *p* = .05. Additionally, the STN group showed a main effect of Stimulation (Table 2), reflecting higher RTs for OFF versus ON.

**TABLE 2:** ANOVA results for measures of Response Time

	NO group					STN group					GPi group				
	*F*	df	*p*‐val	*ηp* ^2^	*ε* ^a^	*F*	df	*p*‐val	*ηp* ^2^	*ε* ^a^	*F*	df	*p*‐val	*ηp* ^2^	*ε* ^a^
Cue	8.73	2,22	.002	0.44	—	12.40	1.3,16.5	.002	0.49	0.64	6.91	2,12	.010	0.54	—
Stimulation	—	—	—	—	—	4.49	1,13	.046	0.27	—	1.06	1,6	.343	0.15	—
Cue a stimulation	—	—	—	—	—	0.86	1.2,16.0	.389	0.06	0.61	0.56	2,12	.585	0.09	—

^a^ Greenhouse–Geisser Epsilon reported for effects violating sphericity assumption and corrected using Greenhouse–Geisser estimates of sphericity.

Because the Cue x Stimulation interaction effect was non‐significant in both groups, and because it was our effect of interest, we conducted Bayesian statistics using the *jsq* module in Jamovi, in order to assess the evidence in favour of the null hypothesis. By entering the main effects as nuisance variables (Wagenmakers et al., 2018), we could observe moderate evidence in support of the null hypothesis (STN: BF01 = 5.1; GPi: BF01 = 3.3). Table S2 provides additional between‐groups analyses comparing the results of the NO group with both DBS groups separately when the stimulator is ON. The results show no difference and thus, are consistent with the view that DBS has limited impact on proactive inhibition.

### 3.3 Neurophysiological results

#### 3.3.1 NO group

**FIGURE 3:**
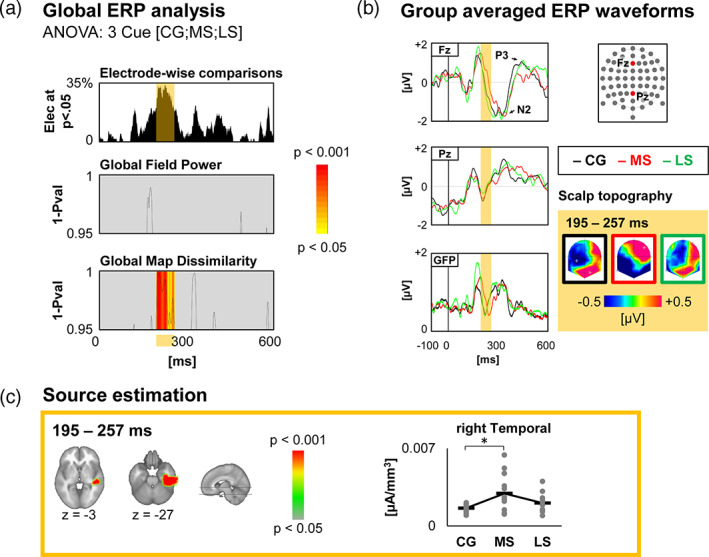
Neuroimaging results for the NO group. (a) Global statistics over the Global Field Power and the Global Map Dissimilarity, with curves indicating the significant time‐points (1‐*p* value) for the main effect of Cue. The yellow to red colour scale represents the *p*‐values for periods satisfying the duration threshold. The electrode‐wise comparison graph represents for each time‐point the percentage of electrodes showing a significant main effect of Cue. (b) Waveforms of two electrodes (Fz and Pz) and the Global Field Power for each condition. Scalp topographies for each condition, averaged over all time‐points of the period of significance over the Global Map Dissimilarity. (c) Results of source estimation for the period of significance. The graph on the right depicts current densities. Thick horizontal lines represent the mean, and the grey dots represent the individual data. Holm‐Bonferroni corrected post‐hoc *t* tests detailing the main effects of Cue are shown (*p < .05)

At the scalp level, the one‐way repeated measures ANOVA (Cue [CG; MS; LS]) on GMD revealed a period of topographic dissimilarities between 195 and 257 ms post‐cue signal, and no periods of difference for GFP (Figure 3a,b). Source estimation localised this difference within the right temporal areas. In this area, current density was higher following MS cues compared to CG and LS cues (Figure 3c). However, the MS versus LS post‐hoc test did not survive the Holm‐Bonferroni correction (*p* = .048 uncorrected). Detailed source estimation results can be found in Table S3.

#### 3.3.2 STN group

**FIGURE 4:**
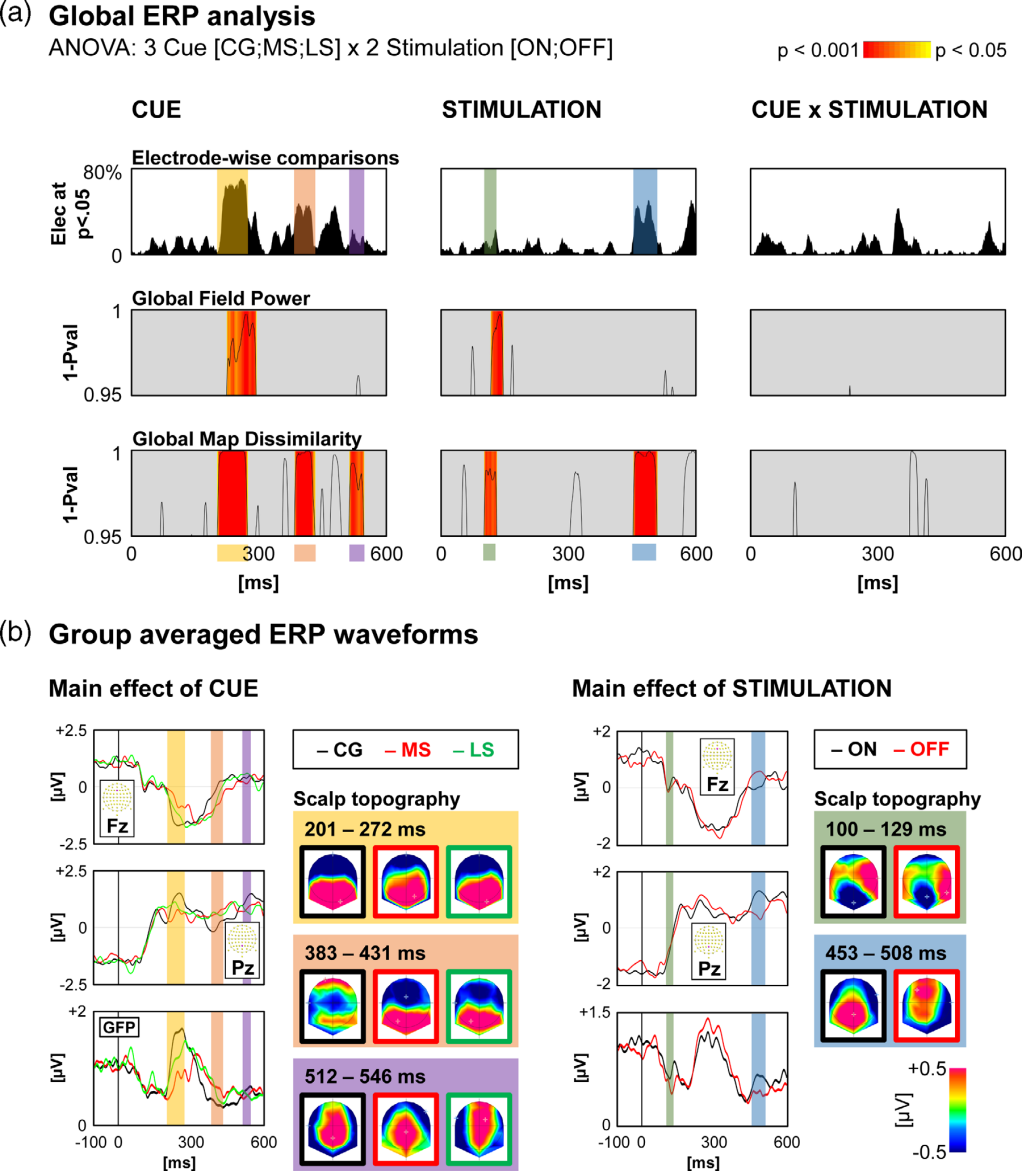
Scalp level neuroimaging results for the STN group. (a) Global statistics over the Global Field Power and the Global Map Dissimilarity, with curves indicating the significant time‐points (1‐*p* value). The yellow to red colour scale represents the *p*‐values for periods satisfying the duration threshold. The electrode‐wise comparison graph represents for each time‐point the percentage of electrodes showing a significant effect. (b) Waveforms of two electrodes (Fz and Pz) and the Global Field Power for each contrast showing significant effects. Scalp topographies for each condition, averaged over all time‐points of each period of significance over the Global Map Dissimilarity for the main effects of Cue and of Stimulation

At the scalp level, the repeated measures ANOVAs (Stimulation [ON; OFF] × Cue [CG; MS; LS]) on GMD revealed three periods of main effect of Cue, and two periods of main effect of Stimulation (Figure 4).

**FIGURE 5:**
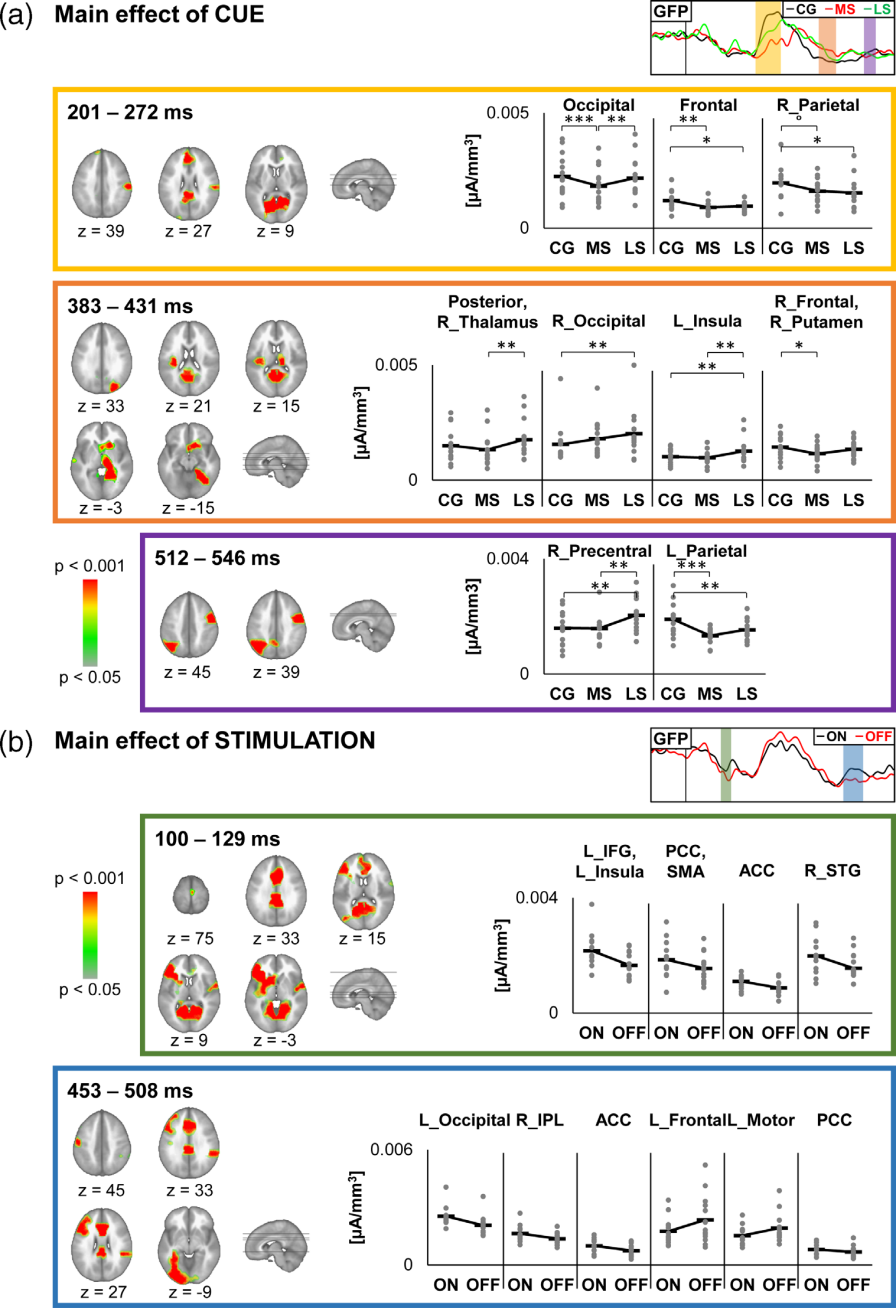
Source estimation results for the STN group. Results of source estimation for each periods of significance for main effects of Cue (a) and of Stimulation (b). The graph depicts current densities. Thick horizontal lines represent the mean, and the grey dots represent the individual data. Holm‐Bonferroni corrected post‐hoc *t* tests detailing the main effects of Cue are shown (***p < .001; **p < .01; *p < .05; °*p* = .052)

Regarding the main effect of Cue, the first period (201–272 ms), was accompanied by a corresponding period of significant GFP effect (223–293 ms). Source estimation localised this difference within the bilateral occipital cortex, the bilateral medial frontal gyrus, and the right inferior parietal lobule. The occipital cluster showed lower current density following MS cues compared to CG and LS cues. The frontal and parietal clusters showed higher current density following CG cues compared to MS and LS cues (Figure 5a). Detailed source estimation results can be found in Table S4.

Source estimation for the second period (383–431 ms) localised the difference centred around bilateral posterior areas, the thalamus, the left insula, and the right orbitofrontal gyrus and putamen (Table S4). The posterior and thalamus cluster, and the insula cluster showed stronger activation following LS cues as compared to CG and MS cues. The occipital cluster showed a gradual increase in activation as the probability of having a NoGo signal increased. The frontal and basal ganglia cluster showed stronger activity following CG cues as compared to MS cues (Figure 5a).

Source estimation for the third period (512–546 ms) localised the difference within the right precentral gyrus, and the left inferior parietal lobule. The precentral cluster showed stronger activation following LS cues as compared to CG and MS cues. The parietal cluster showed stronger activation following CG cues as compared to MS and LS cues (Figure 5a).

Regarding the main effect of Stimulation, source estimation for the first period (100–129 ms) localised the difference within the bilateral anterior cingulate cortex, posterior cingulate cortex, and supplementary motor area, the left inferior frontal gyrus and insula, and the right superior temporal gyrus (Table S4). For all clusters, activation was stronger in the ON versus OFF condition (Figure 5b).

Source estimation for the second period (453–508 ms) indicated stronger activation in the ON versus OFF condition within the bilateral anterior and posterior cingulate cortices, the left occipital cortex, and the right inferior parietal lobule (Table S4). Activation was stronger in the OFF versus ON condition within the left frontal and motor cortices, while the other areas showed stronger activity in the ON versus OFF condition (Figure 5b).

#### 3.3.3 GPi group

**FIGURE 6:**
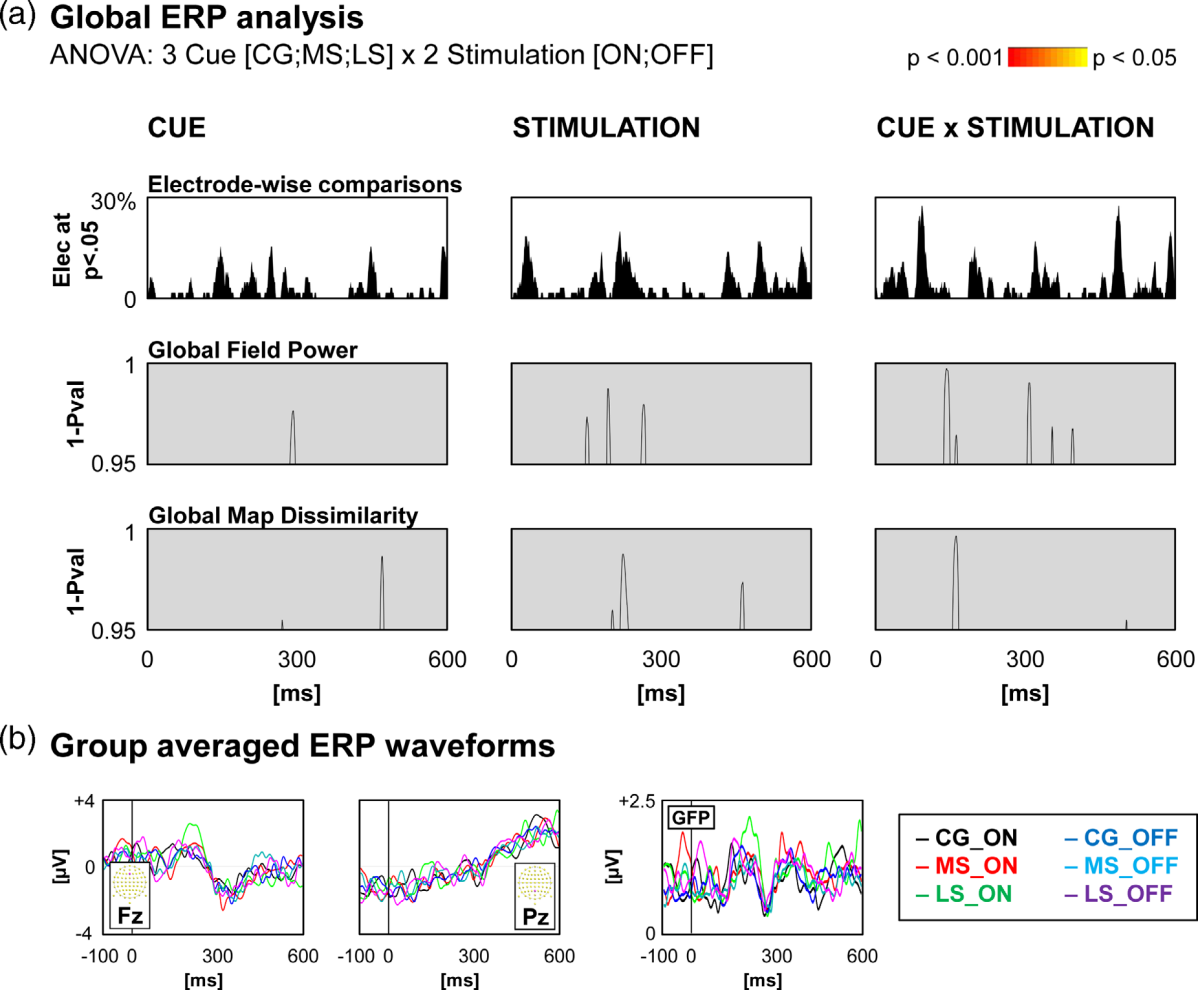
Scalp level neuroimaging results for the GPi group. (a) Global statistics over the Global Field Power and the Global Map Dissimilarity, with curves indicating the significant time‐points (1‐*p* value). No period of significance reached the duration threshold. The electrode‐wise comparison graph represents for each time‐point the percentage of electrodes showing a significant effect. (b) Waveforms of two electrodes (Fz and Pz) and the Global Field Power for each condition

Analysis in the GPi group showed no periods of significant difference for neither GFP nor GMD (Figure 6).

## 4 DISCUSSION

We aimed at characterising the functional correlates of proactive inhibition. We examined the effects of the electrical stimulation of the STN and GPi subcortical structures on proactive inhibitory control, and its electrophysiological correlates with ERP during a cued go/no‐go task. Behaviourally, we found no specific effect of either STN or GPi DBS on measures of proactive inhibition, despite an improvement with DBS ON of motor performance in the STN group, as indexed by reaction times reduction. The absence of a behavioural effect was confirmed by Bayesian analysis and reflected in the EEG results.

However, similarly to the NO group, the STN group showed modulations of EEG topographies (independently from the stimulation condition) depending on the preparedness to stop. In this group, source localisation suggests the involvement of an attentional network and of a right lateralized fronto‐basal network, the latter more specifically involved with proactive inhibition, and compatible with the recruitment of the indirect pathway. A later activation of the hyperdirect pathway is made possible by the activation of the right precentral gyrus.

### 4.1 No evidence of DBS effects on proactive inhibition

Based on previous models, we expected that both STN and GPi DBS could decrease an excessive ‘tonic’ proactive inhibition, resulting in motor facilitation (Aron et al., 2007b; Jahanshahi et al., 2015; Jahanshahi & Rothwell, 2017). For both targets, the effects were difficult to predict, but we speculated that an excessive proactive inhibition is restored to normal levels by DBS. However, we predicted an impairment of the capacity to adapt the restraint on the likelihood of a stop signal to come. Depending on the main circuit involved, we anticipated a different magnitude of this effect. If proactive inhibition is mainly regulated by the hyperdirect pathway, a stronger effect should be observed after STN versus GPi‐DBS. Alternatively, the modulation of proactive inhibitory control occurs through the indirect pathway – in this case the effects would be more readily caused by GPi‐DBS.

Although our predictions were based on theoretical models and on (limited) previous experimental evidence, we could find no effect of DBS on proactive inhibition at the behavioural level. The absence of any effect could be explained by three main reasons: insufficient statistical power, experimental task design, and model inaccuracies.

Collecting sufficient data from clinical populations is always challenging, particularly so when the inclusion criteria are stringent and the suitable population is limited, as is the case in studies on DBS patients. It is therefore likely that increasing the number of patients could have unveiled more robust results, but the Bayesian analyses performed seem to confirm the validity of our results. Moreover, our cohort is similar to or larger than previous studies addressing this topic (Kohl et al., 2015; Mirabella et al., 2013; Obeso et al., 2013).

Our findings are in line with previous experimental work, which did not find significant effects either (Mancini et al., 2019; Mirabella et al., 2012). Proactive inhibition is a complex function, and our (simple) task probably does not capture all the involved components. For instance, the timing of proactive inhibition implementation and release appears to be particularly important. In Parkinson's disease (PD), plan updating of a prepared motor action occurs in part after movement initiation, perhaps due to deficits in proactive inhibition release (Leis et al., 2005). STN‐DBS appears to restore this function to more physiological levels (Mirabella et al., 2013).

A recent paper could demonstrate that STN‐DBS induces a longer stopping time of a continuous ongoing movement, a different approach than testing the suppression of planned, incumbent actions before their execution, as it is normally the case in SSRT or GNG tasks (Lofredi et al., 2020). In future experiments, it will be interesting to test whether this effect occurs through an impairment of proactive inhibition.

Although our task was not designed to capture the release of proactive inhibition during an ongoing movement (Lofredi et al., 2020), or after movement initiation (Leis et al., 2005; Mirabella et al., 2013), we are confident it recorded the most determinant aspects of the cognitive mechanisms underlying the preparation to movement inhibition. The validity of our behavioural findings is strengthened by the results of the EEG analyses, as we found no interaction between the stimulation and proactive inhibition conditions, in both DBS groups.

DBS is thought to remove the antikinetic effect of STN glutamatergic projections by inactivating neuronal somata as in a functional lesion (Chiken & Nambu, 2016). According to the model proposed by Frank (Frank, 2006; Wiecki & Frank, 2013), one of STN functions would be to send a general ‘stop’ signal to ongoing motor programs, in the presence of conflicting external stimuli. From a cognitive perspective, it would ‘buy time’ for other structures to elaborate appropriate responses to external stimuli (‘hold your horses’). The sudden increase of STN antikinetic activity and the modulation of its threshold are thought to be key in reactive and proactive inhibition, respectively.

Areas of STN mediating inhibition are likely far from the electrode, and only slightly affected by the stimulation. This assumption is in line with the known organisation of STN, which is constituted by sub‐areas with different functional specialisation (Accolla et al., 2016). We speculate that the associative areas of STN are more involved in motor inhibition than pure motor areas, which are the target of DBS electrode implantation. This has been recently demonstrated in a task during intraoperative recordings (Mosher, Mamelak, Malekmohammadi, Pouratian, & Rutishauser, 2021). This is also compatible with the observation that DBS impairs motor inhibition only in high conflict situations (Georgiev, Dirnberger, Wilkinson, Limousin, & Jahanshahi, 2016) or under speed pressure (Pote et al., 2016), and that there is a role for the electrode localisation (more ventral within the STN; Hershey et al., 2010; Rodriguez‐Oroz et al., 2011). A recently described direct connection between IFC and ventral STN confirms this interpretation (Chen et al., 2020). We did not reconstruct the electrode localisation due to a limited access to imaging for this project, but we assume that stimulating contacts were located in the latero‐dorsal STN, given the preoperative planning and the good clinical outcome of the intervention (Horn, Neumann, Degen, Schneider, & Kühn, 2017).

### 4.2 STN stimulation enhances attentional processes

We observed an improvement of RT under the STN‐DBS condition, compared to the OFF state. However, this improvement occurred with a comparable magnitude in all cue conditions, and was thus not related to an effect of DBS on proactive inhibition mechanisms. We interpret this finding as a general motor improvement effect. Note that our task did not allow for differentiating reaction times from movement times.

At the EEG level, we found two epochs of effect of stimulation alone after cue presentation. The source localisation shows that this early effect is strongly left lateralised. This localization is compatible with the fact that in our protocol, we turned the stimulator ON and OFF only on one side (the left one for all except one left‐handed patient).

The first effect occurred very early, at 100–120 ms after cue presentation a latency probably corresponding to modulation of primary sensory or attentional processes. We could speculate that STN DBS promotes an increased reactivity immediately after cue presentation (unrelated to the semantic content of the cue), and maybe that this translates into a faster motor response later on: source localisation was consistent with a brain executive attention network called ‘cingulo‐opercular network’ in fMRI studies (Neta, Nelson, & Petersen, 2017), involving the anterior cingulate and anterior insula.

At a latency of 450 ms, a second effect of STN stimulation may be related to a preparatory phase of task execution, but unrelated to cue meaning. This activity could be related to the beginning of the expectation of the probe presentation, to the preparatory phase of decision making, or both. At this latency, left frontal and motor areas were more strongly activated in the OFF condition, when response times were slower. This might be explained by the detrimental effect of over‐anticipating the upcoming signal (De Pretto, Sallard, & Spierer, 2016).

### 4.3 Internal globus pallidus (GPi)

We did not obtain any relevant results in the GPi‐DBS population, which of course does not exclude a role of this structure in proactive inhibition, neither a related behavioural effect of GPi‐DBS. While the limited number of patients in this group call for a cautious interpretation, our findings suggest that STN DBS does not affect proactive inhibition mechanisms, and this is probably true for GPi‐DBS as well.

From a clinical point of view, our observations are rather reassuring in terms of the safety of both STN and GPi‐DBS because they confirm that the stimulation does not impair proactive inhibition capacities.

### 4.4 Electrical neuroimaging: A proactive inhibition network? Main effects of cue

In the STN group, we found a main effect of cue at three different time windows after cue presentation, at 200–270 ms, 380–430 ms, and 510–550 ms, respectively. A topographic ERP modulation very similar to the first one is also observed in the non‐operated group (200–260 ms). This finding gives important insights into the neurophysiology of proactive inhibition. The fact that the source estimation results do not align with the behavioural results (e.g., gradual increase in reaction time, while at the current density level, the MS condition is often at odds with the other two conditions) reflects the fact that if current densities represent engagement of a given area, behavioural performance are the result of all brain processes at once. Additionally, we must highlight that here, ERPs are time‐locked to the cue and thus reflect processing of the Cue meaning and/or anticipation of the response, and not the motor execution of the command itself.

### 4.5 Main effect of cue (STN: 200–270 ms; NO: 200–260 ms)

At around 200 ms, both the NO and the STN groups showed that the ‘maybe stop’ condition differed from the ‘certainly go’ and ‘likely stop’ conditions. In the NO group, scalp topographies and ERP waveforms indicate a delay in latency for the MS condition at the beginning of the N2‐P3 complex (frontal to posterior positivity; Figure 3b). In the STN group, the difference lies fully within the N2 component (frontal negativity, Figure 4b). However, the ERP waveforms indicate a lower amplitude (or latency delay) in the MS condition. Thus, even though the source estimations are different, the effects observed in both groups most likely reflect the same processing. The right temporal effect observed in the NO group might be related to the fact that the CG and LS time windows are at the start of N2.

In reactive inhibition studies (response to a Go or NoGo stimulus), the N2 and P3 components have been associated with conflict monitoring and decision making (Falkenstein, 2006; Hartmann, Sallard, & Spierer, 2016; Manuel, Grivel, Bernasconi, Murray, & Spierer, 2010; Verbruggen & Logan, 2015). Earlier latencies have been associated with improved neural efficiency after training (Benikos, Johnstone, & Roodenrys, 2013), and may reflect a better association between stimulus and response (De Pretto et al., 2017; Spierer, Chavan, & Manuel, 2013). Here, it is possible that the MS cue was considered by the participants as most uncertain regarding the upcoming Go or NoGo stimulus, increasing the time to decide whether to anticipate a go or an inhibition response. Indeed, even though the probability of having a NoGo stimulus after an MS cue was the same as the probability of having a Go stimulus after an LS cue, the participants might have considered the MS cue as an in‐between, uncertain, condition. This interpretation remains highly speculative, as unfortunately we did not ask them feedback questions on how they perceived the cues. In the STN group however, the parieto‐occipital cluster that distinguishes MS from CG and LS has been involved in visual and attentional processes (Spay et al., 2018), and fits with this interpretation.

### 4.6 Direct, indirect and hyperdirect pathway in proactive inhibition

It is worth underscoring that localised source estimation within deep brain structures must be interpreted with caution (Cohen, Cavanagh, & Slagter, 2011; Seeber et al., 2019). Even more so given the inhomogeneity of our population. We tentatively interpret our results within the framework of known cortico‐subcortical circuitry.

At around 400 ms (380–430 ms) after cue presentation, the presence or absence of proactive inhibition per se could play a role in the observed recordings. At this moment, in the CG condition, and to a lesser extent in the LS condition the P3 component is almost at its peak, whereas it still lies at the transition between N2 and P3 following the MS cues (Figure 4b). This shift in latency is consistent with our interpretation of MS being more indecisive. The P3 component has been associate with the implementation of the inhibition command (De Pretto et al., 2017; Spierer et al., 2013). Here, it might reflect proactive inhibition mechanisms, such as best illustrated by the right superior (temporo‐) occipital cluster. This cluster showed an increase in current density as the probability of a NoGo signal increases. Similarly, the posterior‐thalamic cluster and the left insula showed stronger activation in the LS conditions. Within the right fronto‐basal cluster, activation was stronger in the CG condition.

At around 510–550 ms, an additional significant peak was found. Its topography was consistent with an increased activation of right pre‐central gyrus in the LS condition, and of a left parietal region in the CG condition (Figure 5a).

As stated above, subcortical source localization must be interpreted with caution and likewise, our conclusions about involved pathways or circuits definitely need further confirmation. We here propose that the peak at around 400 ms from cue presentation corresponds to the engagement of the indirect pathway in proactively adjusting inhibition reactivity, in preparation for the upcoming probe signal. The higher activation of the right putamen, possibly of the right caudate and of the thalamic regions in the CG vs MS condition (Figure 5a, orange panel) could be interpreted as an early release of proactive inhibition, assuming striatum as a globally prokinetic structure within the direct pathway. Later on, at around 500 ms, the hyperdirect pathway mediates a further release of inhibition, by reducing the relative activity in the right precentral gyrus: the pattern of activation is consistent with a lower activity in conditions when the probability of a Go signal, thus of an upcoming movement, was higher.

The cluster located in the right temporo‐occipital region (peak at around 400 ms) and in the left parietal regions (~500 ms) are harder to interpret, but could be related to anticipated movement planning (Manuel et al., 2010).

Previous studies have not identified specific brain areas responsible for governing proactive inhibition. Most of the evidence suggests that proactive inhibition acts through the modulation of the reactive inhibition network, pre‐SMA and SMA being the most likely areas tuning its activation threshold (Stuphorn & Emeric, 2012). A recent fMRI study with dynamic causal modelling (DCM) analyses attempted at differentiating among reactive and proactive inhibition (Zhang & Iwaki, 2019). A network involving right DLPFC, left caudate, and right IFG was found to be more specifically related to proactive inhibition. On the contrary, a network involving IFG – SMA – STN – M1 was a common pathway shared by both inhibition modalities. Although not conclusive, our data seem to confirm the central role of the indirect pathway in proactive inhibitory control, the direct and hyperdirect pathway possibly intervening at a later stage, releasing inhibition immediately before the go/no‐go signal if chances of stopping are low.

Our task was designed to be easily understood and performed by patients, and to specifically unveil a prolongation of reaction times linked to the presence of proactive inhibition. It consistently succeeded in capturing this effect in all groups, even though intergroup differences in terms of duration of disease, dopaminergic medication and cognition were – as expected – pronounced in our cohort, and motivated us to analyse groups separately. Despite this premise, several limitations must be acknowledged. In our experimental design, intervention was limited to turning OFF and ON only one stimulation side, the left one in all cases but one. This might have limited our capacity to find relevant behavioural effects of stimulation. However, this choice was dictated by several considerations. We wanted to limit the discomfort caused by the off state, particularly in STN patients; this was also instrumental for keeping populations comparable, as GPi stimulation is often particularly effective on tremor and dyskinesias (Williams et al., 2014) but is less effective on bradykinesia and rigidity, and allows for a lesser reduction of dopaminergic medication (as confirmed in our cohort, see Table 1): a bilateral OFF is milder in GPi‐DBS than in STN‐DBS patients.

Another potential limitation is that our EEG analyses were time‐locked to the cue presentation. Interesting information might be retrieved from analyses of the response‐locked ERPs. However, the signal‐to‐noise ratio of the response‐locked signal might be unbalanced given the short delay between the Go signal and the response, and the variable response times depending on the Cue.

The similar behavioural results associated with dissimilar ERP results might suggest unreliable EEG results. However, to our view, this phenomenon is better explained by different brain processes, either due to different neurophysiological mechanisms, or due to different strategies. Regarding the ERPs, the NO group, for which the duration of the disease is much shorter than the other groups, and which does not have DBS implanted, shows average ERPs (Figure 3) very similar to what might be observed in healthy participants (Angelini et al., 2016; De Pretto, Hartmann, Garcia‐Burgos, Sallard, & Spierer, 2019). In the STN group, the ERP components are less pronounced (see Figure 4). However, the topographic maps indicate clear periods of stable brain states.

Results regarding the GPi population should be interpreted with particular caution. This group was difficult to recruit, given that this target is rarely chosen for implantation. Moreover, among the reasons to choose GPi, some cognitive and psychiatric considerations might have been taken into account at the time of surgery, which may influence both the intergroup and the GPi within‐group subject variability (Figure 6). Thus, the absence of a Cue effect in the GPi group may be related to a lack of power due to the low number of participants, as illustrated by the noisy average ERPs.

Finally, the small number of trials is probably the main limiting factor affecting the robustness of our results. A pilot phase with longer experiment duration was rapidly interrupted when it was clear that patients fatigue grossly impaired performance, particularly in the GPi group.For most of our patients, the task was performed with the dominant hand (the right one in all cases but one), while turning on and off the stimulator only on the contralateral side. This means that the right STN was for most of the subjects always stimulated. The inhibition network is thought to be at least partially right lateralized (Aron & Poldrack, 2006; Lofredi et al., 2020). However, recent experimental work shows that the effects on inhibition are only observed when bilateral stimulation is active (Mancini et al., 2019).

## 5 CONCLUSIONS

Our results suggest that proactive inhibitory control has dedicated brain networks, distinct from those governing reactive inhibition. Together with previous evidence, our findings support the hypothesis that the indirect pathway is the main involved circuit, and that the hyperdirect pathway has likely a secondary role. Further studies are needed to confirm this interpretation, also considering that our electrical neuroimaging approach is relatively novel in this field and needs further confirmatory studies.

Despite being remarkably effective on the motor symptoms of Parkinson's disease, both STN and GPi stimulation do not appear to interfere with the mechanisms responsible for adapting the threshold of motor inhibition deployment. Our findings confirm the general safety of DBS, with few effects on executive functions despite the remarkable motor improvement. Future research work will be tasked to investigate if proven behavioural side effects of DBS ‐ mostly caused by electrode misplacement (i.e., hypomania) are accompanied by impairment of proactive inhibitory control.

## CONFLICT OF INTEREST

The authors declare that they have no competing interests.

## ETHICS APPROVAL

The protocol was approved from the local ethics committee (Protocol PB_2016‐01384).

## PATIENT CONSENT

All patients signed an informed consent according to the Declaration of Helsinki.

## Supporting information


**Supplementary Table S1** Behavioural results
**Supplementary Table S2** Between‐group ANOVA results for measures of response time
**Supplementary Table S3** Main brain areas showing significant effects for the NO group
**Supplementary Table S4** Main brain areas showing significant effects for the STN group

## Data Availability

The data that support the findings of this study are available on request from the corresponding author. The data are not publicly available due to ethical restrictions.
