# Supplementary material for: Proactive inhibition is not modified by deep brain stimulation for Parkinson's disease: An electrical neuroimaging study
Source: Hum Brain Mapp. 2021 Jun 10;42(12):3934–49. doi: 10.1002/hbm.25530 (PMC8288097; doi:10.1002/hbm.25530)
Supplement: Supplementary file 1 — Supplementary Table S1 Behavioural results Supplementary Table S2 Between‐group ANOVA results for measures of response time Supplementary Table S3 Main brain areas showing significant effects for the NO group Supplementary Table S4 Main brain areas showing significant effects for the STN group [file HBM-42-3934-s001.docx]

| **Supplementary Table 1 – Behavioural results** | | |
| --- | --- | --- |
| **Condition** | **RT** | **FA** |
|  | Mean (Std) | Mean (Std) |
|  | [ms**]** | [%] |
| *NO GROUP* |  |  |
| CG | 314 (78) | - |
| MS | 347 (77) | 31.5 (24.1) |
| LS | 401 (59) | 7.1 (9.5) |
|  |  |  |
| *STN GROUP* |  |  |
| CG_ON | 343 (107) | - |
| MS_ON | 385 (78) | 13.5 (17.5) |
| LS_ON | 455 (67) | 9.5 (10.2) |
| CG_OFF | 376 (110) | - |
| MS_OFF | 420 (80) | 19.0 (18.2) |
| LS_OFF | 469 (92) | 5.0 (5.2) |
|  |  |  |
| *GPi GROUP* |  |  |
| CG_ON | 337 (111) | - |
| MS_ON | 386 (118) | 14.3 (16.6) |
| LS_ON | 424 (94) | 6.9 (6.6) |
| CG_OFF | 338 (94) | - |
| MS_OFF | 379 (76) | 14.3 (10.6) |
| LS_OFF | 400 (69) | 7.4 (6.8) |
| CG: Certainly Go condition; LS: Likely Stop condition; MS: Maybe Stop condition | | |

| **Supplementary Table 2 –  Between-group ANOVA results for measures of Response Time** | | | | | | | | | | | |
| --- | --- | --- | --- | --- | --- | --- | --- | --- | --- | --- | --- |
|  | **NO vs STN** | | | | |  | **NO vs GPi** | | | | |
|  | F | df | p-value | ηp^2^ | ε* |  | F | df | p-value | ηp^2^ | ε* |
| Cue | 22.95 | 2,1.37 | <0.001 | 0.47 | 0.68 |  | 13.06 | 2,1.54 | <0.001 | 0.54 | 0.77 |
| Group | 2.79 | 1,26 | 0.107 | 0.10 | - |  | 0.70 | 1,19 | 0.413 | 0.04 | - |
| Cue * Group | 0.60 | 2,1.37 | 0.50 | 0.02 | 0.68 |  | 0.26 | 2,1.54 | 0.717 | 0.01 | 0.77 |
| * Greenhouse-Geisser Epsilon reported for effects violating sphericity assumption and corrected using Greenhouse-Geisser estimates of sphericity | | | | | | | | | | | |

| **Supplementary Table 3 –  Main brain areas showing significant effects for the NO group** | | | | | | | |
| --- | --- | --- | --- | --- | --- | --- | --- |
| **Brain region** | **Hemisphere** | **x** | **y** | **z** | **F-values** | **p-value** | **Cluster size** |
| *Period 1: 195 - 257 ms* |  |  |  |  |  |  |  |
| Superior Temporal Gyrus | Right | 51 | -27 | -3 | 6.71 | 0.005 | 121 |
| Middle Temporal Gyrus | Right | 63 | -27 | -27 | 6.24 | 0.007 |  |
| *Note:* MNI x y z coordinates of local maxima for each significant cluster at *p* < .05. Minimal cluster size: 19 solution points. Brain areas are grouped by cluster. | | | | | | | |

| **Supplementary Table 4 –  Main brain areas showing significant effects for the STN group** | | | | | | | |
| --- | --- | --- | --- | --- | --- | --- | --- |
| **Brain region** | **Hemisphere** | **x** | **y** | **z** | **F-values** | **p-value** | **Cluster size** |
| CUE |  |  |  |  |  |  |  |
| *Period 1: 201 - 272 ms* |  |  |  |  |  |  |  |
| Cuneus | Left | 3 | -69 | 9 | 9.22 | 0.001 | 276 |
| Cuneus | Right | 21 | -69 | 3 | 9.18 | 0.001 |  |
| Medial Superior Frontal Gyrus | Left | -9 | 45 | 27 | 8.27 | 0.002 | 59 |
| Inferior Parietal Lobule | Right | 63 | -21 | 39 | 4.89 | 0.016 | 24 |
|  |  |  |  |  |  |  |  |
| *Period 2: 383 - 431 ms* |  |  |  |  |  |  |  |
| Posterior Cingulate Cortex | Left | -3 | -57 | 15 | 6.21 | 0.006 | 152 |
| Inferior Occipital Gyrus | Right | 39 | -63 | -9 | 5.75 | 0.009 |  |
| Thalamus | Right | 15 | -33 | 3 | 4.49 | 0.021 |  |
| Superior Occipital Gyrus | Right | 33 | -87 | 33 | 6.15 | 0.006 | 27 |
| Insula | Left | -39 | -21 | 21 | 4.97 | 0.015 | 24 |
| Orbitofrontal Gyrus | Right | 21 | 27 | -15 | 4.11 | 0.028 | 54 |
| Putamen | Right | 27 | 21 | -3 | 4.02 | 0.030 |  |
|  |  |  |  |  |  |  |  |
| *Period 3: 512 - 546 ms* |  |  |  |  |  |  |  |
| Precentral Gyrus | Right | 51 | -3 | 45 | 11.79 | 0.000 | 58 |
| Inferior Parietal Lobule | Left | -45 | -63 | 39 | 7.86 | 0.002 | 126 |
|  |  |  |  |  |  |  |  |
| STIMULUS |  |  |  |  |  |  |  |
| *Period 1: 100 - 129 ms* |  |  |  |  |  |  |  |
| Inferior Frontal Gyrus | Left | -51 | 39 | 15 | 30.64 | 0.000 | 301 |
| Insula | Left | -39 | -9 | -9 | 10.20 | 0.007 |  |
| Posterior Cingulate Cortex | Right | 9 | -57 | 9 | 15.69 | 0.002 | 308 |
| Posterior Cingulate Cortex | Right | 9 | -33 | 45 | 7.70 | 0.016 |  |
| Supplementary Motor Area | Right | 3 | -15 | 75 | 5.22 | 0.040 |  |
| Anterior Cingulate Cortex | Right | 9 | 27 | 33 | 10.84 | 0.006 | 103 |
| Superior Temporal Gyrus | Right | 63 | 3 | -3 | 7.62 | 0.016 | 39 |
|  |  |  |  |  |  |  |  |
| *Period 2: 453 - 508 ms* |  |  |  |  |  |  |  |
| Middle Occipital Gyrus | Left | -15 | -93 | -9 | 15.83 | 0.002 | 165 |
| Inferior Occipital Gyrus | Left | -33 | -81 | -9 | 15.03 | 0.002 |  |
| Inferior Parietal Lobule | Right | 51 | -39 | 33 | 15.22 | 0.002 | 27 |
| Anterior Cingulate Cortex | Left | -9 | 27 | 27 | 13.94 | 0.003 | 51 |
| Inferior Frontal Gyrus | Left | -45 | 15 | 27 | 10.11 | 0.007 | 60 |
| Middle Frontal Gyrus | Left | -33 | 51 | 33 | 7.50 | 0.017 |  |
| Primary Motor Cortex | Left | -57 | -9 | 45 | 9.16 | 0.010 | 23 |
| Posterior Cingulate Cortex | Right | 3 | -33 | 33 | 7.26 | 0.018 | 23 |
| *Note:* MNI x y z coordinates of local maxima for each significant cluster at *p* < .05. Minimal cluster size: 19 solution points. Brain areas are grouped by cluster. | | | | | | | |
